# Reducing the risk of nosocomial Hepatitis B virus infections among healthcare workers in Nigeria: a need for policy directive on pre-employment screening and vaccination

**DOI:** 10.11604/pamj.2018.30.133.15954

**Published:** 2018-06-14

**Authors:** Olorunfemi Akinbode Ogundele

**Affiliations:** 1 Department of Community Medicine, University of Medical Sciences, Ondo City, Ondo State, Nigeria

**Keywords:** Healthcare workers, policy, screening, vaccination, hepatitis B vrus

## Abstract

Healthcare workers (HCWs) are at increased risk of contracting Hepatitis B virus (HBV) infection and other vaccine-preventable diseases, especially if they are not protected by immunity derived from previous infection or vaccination. Sub-Sahara Africa countries including Nigeria is reported to have the highest rate of HBV. Vaccination of HCWs is essential in protecting them from acute and chronic sequelae of HBV or any other form of vaccine-preventable diseases; however, HCWs vaccination remains a challenge for many developing countries including Nigeria due to lack of policy directive on pre-employment screening and vaccination. Poor political will and inadequate funding of healthcare in the country also impacts negatively on the implementation of effective pre-employment screening and vaccination programmes needed to protect HCWs. The aim of this opinion paper is to promote policy direction on pre-employment screening and vaccination in other to protect HCWs from nosocomial HBV infection. The most appropriate time perhaps for promoting the importance of employee immunisation is during pre-employment screening. The policy options are either for employers to allocate financial resources towards HCWs pre-employment screening and vaccination or alternatively initiate a programme where new HCWs provide evidence of protection against HBV or other vaccine-preventable diseases specified in the policy directive. Protecting HCWs form nosocomial HBV infection requires well-articulated policy directive, proper implementation, supported by adequate funding and good political will on the part of employers and government.

## Opinion

Hepatitis B virus infection (HBV) in healthcare settings has the potential to cause serious illness and avoidable deaths in staffs and patients especially in low resource countries of sub-Sahara Africa. HBV is a priority occupationally acquired infection that is associated with serious public and personal health consequences. It's documented as the greatest health hazard for healthcare workers (HCWs) in the hospital settings. Mortality is mainly due to the devastating effect of chronic infection, such as liver cirrhosis, liver failure, and liver cancer [1,2]. Each year an estimated 3 million HCWs suffer from percutaneous exposure to blood borne pathogens [3]. The risk of acquiring HBV among HCWs is four times greater than that of the general population [4]. HBV carriage rate in Nigeria is reported to be in the range of 9-39% in the general populace [5] and about 1.5-17% among HCWs [6-8].

**Current state of HCWs vaccination programme**: The World Health Organization has recommended that high-risk groups, including HCWs, be targeted for routine provision of HBV vaccine to protect them from infection. In spite of this recommendation. Twenty-four percent of the HCWs worldwide remain unvaccinated against HBV [9]. Although HCWs are an easily identifiable population in which to implement vaccination strategies, many countries are confronted with the challenges of addressing at-risk target groups [10]. Nigeria like many other countries in sub-Sahara Africa has no clear policy on vaccination against HBV and some other vaccine-preventable diseases among HCWs. The lack of strategies and policy contributes to the poor uptake of HBV vaccination among HCWs. There is a need to shift from merely detecting presence or absence of diseases during pre-employment medical screening to having a policy directive on vaccination programme for HCWs during employment. This opinion paper aims at promoting policy direction on pre-employment screening and vaccination in other to protect HCWs from nosocomial HBV infection. Immunisation is a successful and cost-effective intervention for prevention of disease. It is expected that government or health service providers formulate a comprehensive vaccination strategy for their workforce in order to ensure that all HCWs are made aware of the benefits of immunisation, the consequences to the HCW if they are not immunised, and for a patient who acquires an infection while in the hospital as an inpatient. The most appropriate time perhaps for promoting the importance of employee immunisation is during pre-employment screening. Nigeria is faced with critical shortages of HCWs; despite shouldering many of the infectious diseases burdens of vaccine-preventable diseases in Sub-Sahara Africa. The country is equally confronted with the challenge of emigration of HCWs seeking good quality life abroad. Any form of morbidity and mortality related to chronic diseases, such as HBV infections will potentially contribute to the high attrition among HCWs in the country. While many African countries including Nigeria have implemented universal HBV vaccination for infants there are little or no efforts, at protecting HCWs from nosocomial HBV infection. The major challenge is the lack of policy on pre-employment screening and vaccination of HCWs against HBV and other vaccine-preventable diseases. Other challenges, however, are inadequate funding and lack of political will on HCWs vaccination programme, unlike the universal vaccination programme which is supported by international partners.

**Policy options**: To address this challenges there is a need for policy directive and implementation strategies on pre-employment screening and vaccinations. The policy options are either to initiate a pre-employment screening and vaccination programme that incorporates training of at-risk or high-risk HCWs on infection control and universal basic precaution. This policy option requires that employers allocate financial resources towards HCWs pre-employment screening and vaccination. They also train them on infection control and universal basic precaution. The alternative is to initiate a programme where new HCWs provide evidence of protection against HBV or other vaccine-preventable diseases specified in the policy directive. The employers also ensure that they comply with the requirements of this policy directive at their own expense, prior to appointment. On appointment, however, they would be trained in infection control and universal basic precaution especially the high- risk group. In this option, job advertisement would be done with advice for potential employees on the requirements of the policy directive. Individual vaccination status and evidence of protection will be assessed to determine if HBV screening is required. If not required an evidence of compliance is issued to the concerned HCWs and offered employment. Also of note are exceptional situations such as individuals with contraindications to vaccinations who should be attended to base on the merit of their situation. Either of these policies and strategies if implemented will no doubt ensure that all HCWs are vaccinated on appointment. Previously employed one should be encouraged to comply with the requirements of the policy directive as need may arise. Appropriate policy and implementation strategies will reduce the risk of nosocomial HBV infections among HCWs in low resource settings as obtained in Nigeria and some other sub-Sahara African countries.

## Conclusion

This opinion paper has presented the need to protect HCWs from nosocomial HBV infections. It has also revealed that proper and well-implemented policy directive, supported by adequate funding and good political will on the part of government is crucial in getting HCWs protected.

## Competing interests

The author declares no competing interest.
